# The Impact of Organised Screening Programs on Breast Cancer Stage at Diagnosis for Canadian Women Aged 40–49 and 50–59

**DOI:** 10.3390/curroncol29080444

**Published:** 2022-08-09

**Authors:** Anna N. Wilkinson, Jean-Michel Billette, Larry F. Ellison, Michael A. Killip, Nayaar Islam, Jean M. Seely

**Affiliations:** 1 Department of Family Medicine, Faculty of Medicine, University of Ottawa, Ottawa, ON K1H 8L6, Canada; 2 Centre for Population Health Data at Statistics Canada, Ottawa, ON K1A 0T6, Canada; 3 School of Medicine, University of Limerick, V94 T9PX Limerick, Ireland; 4 Clinical Epidemiology Program, The Ottawa Hospital Research Institute, Ottawa, ON K1H 8L6, Canada; 5 Department of Radiology, The Ottawa Hospital Research Institute, University of Ottawa, Ottawa, ON K1H 8L6, Canada

**Keywords:** breast cancer, screening mammography, stage shift, registries, prevention, recommendations, clinical practice

## Abstract

The relationship between Canadian mammography screening practices for women 40–49 and breast cancer (BC) stage at diagnosis in women 40–49 and 50–59 years was assessed using data from the Canadian Cancer Registry, provincial/territorial screening practices, and screening information from the Canadian Community Health Survey. For the 2010 to 2017 period, women aged 40–49 were diagnosed with lesser relative proportions of stage I BC (35.7 vs. 45.3%; *p* < 0.001), but greater proportions of stage II (42.6 vs. 36.7%, *p* < 0.001) and III (17.3 vs. 13.1%, *p* < 0.001) compared to women 50–59. Stage IV was lower among women 40–49 than 50–59 (4.4% vs. 4.8%, *p* = 0.005). Jurisdictions with organised screening programs for women 40–49 with annual recall (screeners) were compared with those without (comparators). Women aged 40–49 in comparator jurisdictions had higher proportions of stages II (43.7% vs. 40.7%, *p* < 0.001), III (18.3% vs. 15.6%, *p* < 0.001) and IV (4.6% vs. 3.9%, *p* = 0.001) compared to their peers in screener jurisdictions. Based on screening practices for women aged 40–49, women aged 50–59 had higher proportions of stages II (37.2% vs. 36.0%, *p* = 0.003) and III (13.6% vs. 12.3%, *p* < 0.001) in the comparator versus screener groups. The results of this study can be used to reassess the optimum lower age for BC screening in Canada.

## 1. Introduction

Early detection of breast cancer through screening mammography and advances in technology and treatments have led to improved breast cancer (BC) survival [1]. Technology improvements such as breast ultrasound and magnetic resonance imaging have also markedly improved the ability of radiologists to diagnose breast cancer and profound changes in treatment have contributed to a 46% reduction in breast cancer mortality since screening mammography began in 1989 in Canada [2]. Treatment advances for breast cancer may raise questions about the relative importance of screening. Yet despite these advances, BC is still expected to be responsible for a quarter of all new cancer diagnoses in Canadian women and 14% of all cancer deaths in women in 2022 [3]. BC is the leading cause of non-accidental death in women younger than 50, and 30% of life years lost to BC are among women diagnosed in their 40s [4].

Mammographic screening for women in their 40s is contentious. The Canadian Task Force on Preventive Health Care (CTFPHC) recommended against routine screening of women aged 40–49 in their 2011 and 2018 guidelines and suggested that care providers should engage in discussion with these patients around screening [5,6]. The eight randomised controlled trials (RCTs) considered in these guidelines were performed between 1963 and 1991, prior to advances such as digital mammography and trastuzumab, and had screening intervals of up to 33 months [7,8,9,10,11,12,13,14]. Meta-analysis of these eight trials showed a BC mortality reduction of 15–18% for women 40–49 [15]. The Canadian National Breast Screening Study was the only trial that did not show a significant mortality reduction with screening [16]; however, the validity of randomisation and image quality in this trial have since been called into question [17,18]. A meta-analysis of the remaining seven trials showed a BC mortality reduction of 24% for the number of women invited to screen [19], and 29% for the combined five Swedish RCTs for women aged 40–49 at entry into screening [15].

Several observational studies exist which contribute valuable population-level insight and more accurately reflect actual screening practices, such as a yearly screening interval. The Pan Canadian trial involved 2.8 million women in Canadian screening programs over a 20-year period and showed a 44% BC mortality reduction in women aged 40–49 who were screened with mammography [20]. A similar rate of 41% reduction in mortality was seen in Sweden in 550,000 women after 10 years of participation in an organised screening program, as well as a 25% reduction in the rate of advanced cancers [21,22]. 

The goal of BC screening is to detect earlier, more treatable stages of BC. Although some studies postulate that the rate of advanced stage BC has not changed since the introduction of screening in the late 1980s [23,24,25], these studies have been criticised because tumor registry data were not linked to exposure to screening, and the annual increase in BC incidence of 1% from 1950 until 2001 was not accounted for [26,27,28]. It is clear that women in organised breast screening programs are more likely to have a lower-stage disease at diagnosis [29]. Early stage BC typically involves treatments with lower morbidity, including the decreased need for mastectomy, axillary dissection, chemotherapy, and radiation therapy, ultimately providing cost savings [30]. Stage-shift with screen detection has been shown to translate into survival benefits and is not merely a reflection of lead-time bias [31,32].

The relationship between breast cancer screening and overdiagnosis has garnered increasing attention, particularly given the improvements in treatments for breast cancer [33]. A recent review by Yaffe and Mainprize demonstrated that overdiagnosis is better-termed overdetection and may occur in the context of screening when cancers that are slow growing or indolent are detected and would not have surfaced clinically or caused the patient’s death [34]. This may be an important harm from screening if treatment is not tailored to the affected individual. Using Canadian modelling data, Yaffe and Mainprize found that rates of overdetection were much lower than estimated by the CTFPHC and were more likely to occur in older women than younger women due to competing causes of death [34]. A very recent Belgian modelling study similarly confirmed that overdiagnosis was much more likely in older women and found that estimates of overdiagnosis were much more accurate with a follow-up of 10 years or more [35].

BC screening programs for women aged 40–49 are considered most effective when they are organised, (i.e., population-based) and when they include annual reminders [4,36]. Active recruitment is generally employed to achieve a target participation rate of 70%. Opportunistic programs where women are required to take an active role in arranging their screening have been shown to have significantly lower screening rates [37]. Annual screening is important as the growth of BC in premenopausal women is more rapid [38]. Younger women aged 40–49 are more likely to have dense breasts, which among other factors increase the risk of BC [39]. It has been shown that mammography in women with extremely dense breasts is more effective if it is performed yearly [40,41]. 

This study assesses the relationship between Canadian mammography screening practices for women aged 40–49 on the stage of BC at diagnosis in women aged 40–49 and 50–59 years using incidence data from the Canadian Cancer Registry (CCR) and screening information from the Canadian Community Health Survey (CCHS) as well as provincial and territorial screening practices. Variations in jurisdictional screening policies in Canada allow this unique opportunity to evaluate the impact of different programmatic screening policies on the stages of breast cancer at diagnosis in women 40–49 and 50–59 years old. To our knowledge, this work has not been performed before in Canada or elsewhere in another country. 

## 2. Methods

The CCR is a population-based database comprised of data annually collected and reported to Statistics Canada by each provincial and territorial cancer registry. Demographic information regarding the individual diagnosed with cancer, and characteristics of the cancer itself, are available for each new primary case. Cancer-specific information, including stage at diagnosis, is available for common cancers, including BC [42]. Individual provinces and territories have varying practices for screening women aged 40–49, ranging from organised screening programs with annual recall to recommendations against screening in several provinces [43]. The Canadian Community Health Survey (CCHS) is a national cross-sectional survey that allows the determination of screening mammography activity [44] with acknowledged inherent bias [45]. Because health care in Canada is publicly funded, regular screening activity was mostly captured in the population-based screening programs, unless screens were performed outside of the organised programs or recorded as diagnostic mammograms. Although information about the method of detection of BC is not available, combining provincial-level BC data with the presence of organised programs and screening activity allows for a comparison of screening policies on stage at diagnosis in age cohorts 40–49 and 50–59.

This study was a secondary analysis of nationally de-identified data collected by Statistics Canada, and as such, ethics approval was not required. Female BC incidence data were obtained from the CCR file released on 19 May 2021 [46]. This version of the CCR included primary invasive cancer cases diagnosed among Canadian residents from 1992 to 2018, although cases diagnosed in Quebec from 2011 onward had not yet been submitted. The analytic file used followed the multiple primary coding rules of the International Agency for Research on Cancer (IARC) [47]. Full staging data were available from 2010 to 2017. Stage data used collaborative stage; a comprehensive standardised system sponsored by the American Joint Committee on Cancer (AJCC) which is compatible with the other staging systems in use during that period [48]. Unstaged BC cases were excluded from our analysis. There was no information on breast density or ethnicity. 

Results for women aged 40–49 at diagnosis were compared to those for women aged 50–59 to assess the impact of screening policies in younger women. The 50–59 age group was chosen because it is the closest age group to 40–49 for which women in all jurisdictions may undergo regular screening mammography

Provincial and territorial screening practices varied across the country (Table 1). Those jurisdictions with screening programs that allowed women to access BC screening in their 40s by self-referral, and subsequently followed these women with annual recall, were designated as screeners [49,50]. Five screener jurisdictions were identified: Nova Scotia, British Columbia, Alberta, Prince Edward Island, and Northwest Territories. Alberta allowed self-referral in 2007, but by 2012 required a physician referral for the first screen. BC changed from annual to biennial recall in 2014. The other jurisdictions collectively formed the comparator group. Quebec was necessarily excluded from this group due to the absence of incidence data from this province in the CCR for the study period. Manitoba had biennial recall, and after the study period Yukon began to send anual reminder letters. In provinces that required a physician referral, some 40–49-year-old women screened may have had a family history of breast cancer that led to the referral. Data from Statistics Canada’s nationally representative CCHS were used to determine the percentage of women aged 40–49 who reported having a screening mammogram in the previous two years [51]. This yielded screening participation rates that were independent of provincial/territorial screening programs. Despite this, these jurisdictions were not included in the “screener” group based on the a priori definition.

Annual average percent changes in age- and stage-specific female BC incidence rates between 2011 and 2017 were calculated using JoinPoint 4.9.0.0 [52], which fit a piecewise-linear regression model that assumed a constant rate of change in the logarithm of the annual incidence rate. The year 2011 was chosen as the starting point for this trend analysis because it corresponded with the CTFPHC recommendation against screening women aged 40–49 years. Because the incidence of BC is lower in women in their 40s than in their 50s, the relative proportions of BC stages at diagnosis for the period from 2010 to 2017 were compared between screener and comparator jurisdictions, and statistical significance was calculated using z-tests. Standard errors for incidence rates were derived directly from the Poisson distribution, whereas those for proportions were calculated using the Agresti–Coull method [53]. 

A linear regression analysis was used to assess the relationship between self-reported jurisdictional screening percentages for 2012 and BC incidence rates for the period from 2011 to 2013. The analysis was restricted to early stage BC, defined by TMIST (AJCC 8th ed) as stage I, and the most advanced BC as stage IV. This time period encompasses the 2012 two-year time frame for reported mammography. Stage migration in women aged 50–59 was investigated by using provincial screening status for women aged 40–49 and comparing proportions of BC stage at diagnosis for women aged 50–59 in screener and comparator provinces. P-values correspond to two-sided tests of the null hypothesis that there was no difference in stage distribution, with a significance level of 0.05.

## 3. Results

**Figure 1:**
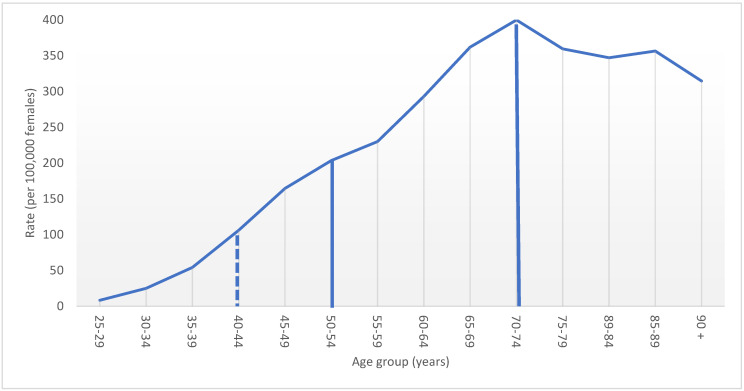
Age–specific female breast cancer incidence rate, by five–year age group, Canada excluding Quebec, 2010 to 2017 period. Note: Quebec is excluded because cases diagnosed in Quebec from 2011 onward had not been submitted to the Canadian Cancer Registry. Solid lines display current ages included in screening; dashed line indicates the age threshold where screening is being explored. Source: Canadian Cancer Registry (1992 to 2018) at Statistics Canada [42].

Female BC incidence rates increased with age until a peak in the 70–74 year age group (Figure 1). Between 2010 and 2017, less than half (46.1%) of all new primary BC cases were diagnosed in women aged 50–74, the age range recommended by current screening guidelines. This percentage increased by almost 9 percentage points to 54.6% when women in their forties are included. The number of BC cases diagnosed in women aged 40–49 was equivalent to 18.5% of the total number of BC cases in screened women aged 50–74. The incidence rate ratio between cases diagnosed in the 40s and those diagnosed in their 50s was 0.63. 

**Figure 2:**
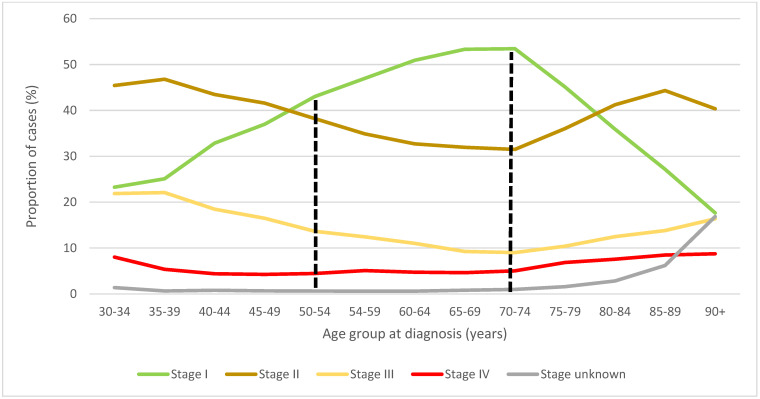
Stage–specific distribution of female breast cancer cases by age group at diagnosis, Canada excluding Quebec, 2010 to 2017 period. Note: Quebec is excluded because cases diagnosed in Quebec from 2011 onward had not been submitted to the Canadian Cancer Registry. The ages between the dashed lines represent those age groups for which mammogram screening is currently recommended. Source: Canadian Cancer Registry (1992 to 2018) at Statistics Canada [42].

The proportion of women diagnosed with stage I BC was observed to be greater among those aged 50–74 at diagnosis—for whom screening is currently recommended—than among those diagnosed outside of this age range (Figure 2). Canadian women aged 50–74 had a proportionally higher incidence of stage I BC, and lower relative proportions of stage II and III BC, compared with younger and older women who fall outside the CTFPHC recommended screening age.

**Figure 3:**
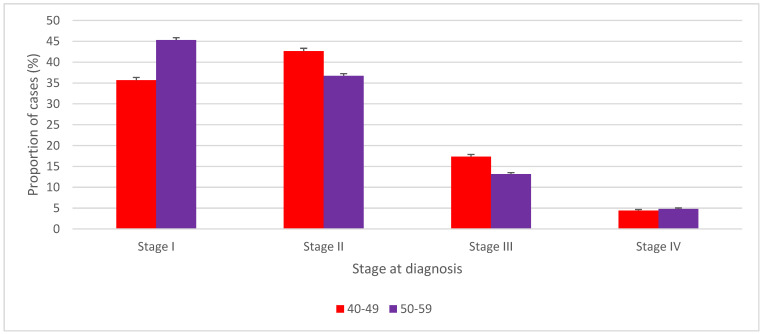
Stage–specific distribution of female breast cancer cases, ages 40 to 49 years versus ages 50 to 59 years, Canada excluding Quebec, 2010 to 2017. Note: Quebec is excluded because cases diagnosed in Quebec from 2011 onward had not been submitted to the Canadian Cancer Registry. Vertical error bars indicate 95% confidence intervals. Source: Canadian Cancer Registry (1992 to 2018) at Statistics Canada [42].

The distribution of BC stage at diagnosis was significantly different between women aged 50–59 and their 40–49 year counterparts not targeted by screening programs (Figure 3, Table 2). Except for stage IV, Canadian women aged 40–49 had BC diagnosed at significantly later stages than those aged 50–59 who had higher relative proportions of stage I BC (45.3% vs. 35.7%; *p* < 0.001), and lower proportions of stage II (36.7% vs. 42.6%, *p* < 0.001), and III (13.1% vs. 17.3%, *p* < 0.001) BC. The proportion of stage IV at diagnosis in women aged 40–49 was significantly lower than in women aged 50–59 years old (4.4% vs. 4.8%, *p* = 0.005).

### 3.1. Screening Participation

From 2003 to 2017, reported BC screening participation generally declined among women aged 40–49, with the largest decrease (21.4 percentage points) occurring in New Brunswick (Table 1). Even in comparator jurisdictions, some screening occurred among women aged 40–49, ranging from 10.0% in Yukon in 2003 to 51.8% in Newfoundland and Labrador in 2008.

### 3.2. Stage Distribution of BC Related to Screening Guidelines for Women 40–49 Years Old

**Figure 4:**
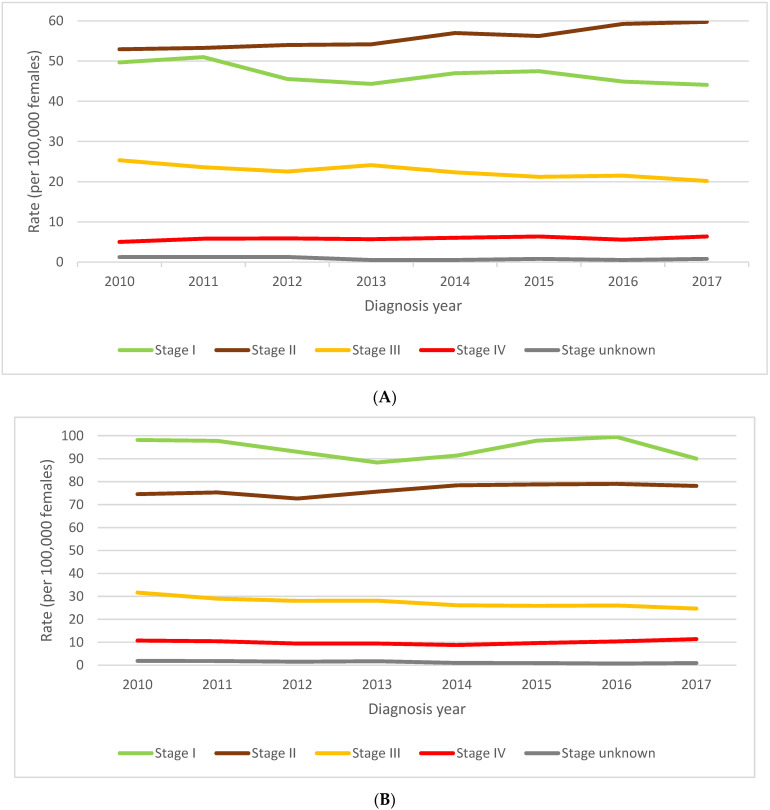
(**A**). Stage-specific female breast cancer incidence rates, ages 40 to 49 years, Canada excluding Quebec, 2010 to 2017. (**B**). Stage-specific female breast cancer incidence rates, ages 50 to 59 years, Canada excluding Quebec, 2010 to 2017. Note: Quebec is excluded because cases diagnosed in Quebec from 2011 onward had not been submitted to the Canadian Cancer Registry. Source: Canadian Cancer Registry (1992 to 2018) at Statistics Canada [42].

**Figure 5:**
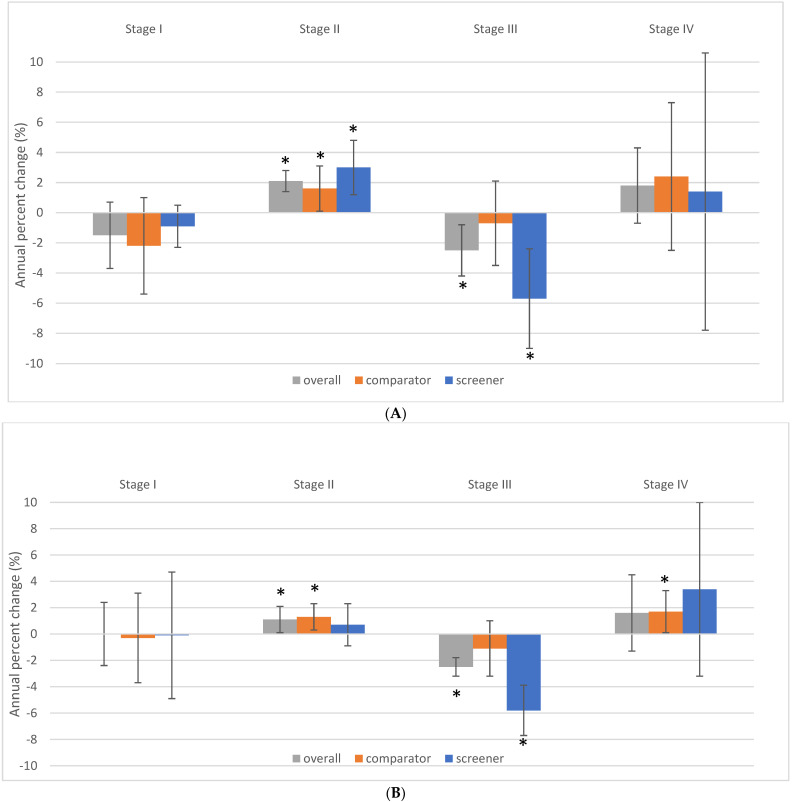
(**A**). Stage–specific female breast cancer incidence rate trends by jurisdictional screening status, ages 40 to 49 years, Canada excluding Quebec, 2011 to 2017. (**B**). Stage-specific female breast cancer incidence rate trends by jurisdictional screening status, ages 50 to 59 years, Canada excluding Quebec, 2011 to 2017. Note: Quebec is excluded because cases diagnosed in Quebec from 2011 onward had not been submitted to the Canadian Cancer Registry. Screeners: Alberta, British Columbia, Nova Scotia, Northwest Territories, and Prince Edward Island; Comparators: Manitoba, New Brunswick, Newfoundland and Labrador, Nunavut, Ontario, Saskatchewan, Yukon. The vertical error bars indicate 95% confidence intervals. Asterisks indicate the trend is significant at the *p* < 0.05 level. Source: Canadian Cancer Registry (1992 to 2018) at Statistics Canada [42]; provincial and territorial screening practices [49,50].

The rate of diagnosis of stage I disease decreased by a non-statistically significant average of 1.5% per year from 2011 to 2017 (*p* = 0.147) for women 40–49 years old (Figure 4 and Figure 5). The corresponding rate of diagnosis of stage II BC in women in their forties increased by an average of 2.1% per year (*p* = 0.001). The increasing trend for stage II cases was more pronounced among screening jurisdictions (3.0% annual average) than among comparators (1.6% annual average). Stage III BC incidence rates decreased by an average of 2.5% per year (*p* = 0.012) over this same time period. This reduction was driven by a 5.7% annual decrease in the rate of diagnosis of stage III cases in screener jurisdictions (*p* = 0.007) (Figure 5A). Stage IV BC rates increased by an annual average of 1.8% per year (*p* = 0.103); 2.4% among comparators (*p* = 0.244) and 1.4% among screeners (*p* = 0.707).

### 3.3. Stage Distribution of BC in Women 50–59 Related to Screening Guidelines for Women 40–49 Years Old

The incidence of stage I BC remained stable in women aged 50–59 over the same time period from 2011 to 2017 (Figure 4B and Figure 5B). A significant average annual increase of 1.1% in the rate of diagnosis of stage II BC was observed (*p* = 0.047). Among stage III BC cases, there was a significant average decline of 2.5% per year (*p* < 0.001) which was mainly influenced by an annual reduction of 5.8% per year in the screener jurisdictions (*p* = 0.001). The overall trend for stage IV was not significant, but there was a significant average annual increase of 1.7% in metastatic disease among comparator women 50–59 (*p* = 0.025). This corresponded to a 10.3% increase in stage IV BC in these women over the six years. 

### 3.4. Impact of Provincial/Territorial Screening Status on BC Stage at Diagnosis in Women Aged 40–49

**Figure 6:**
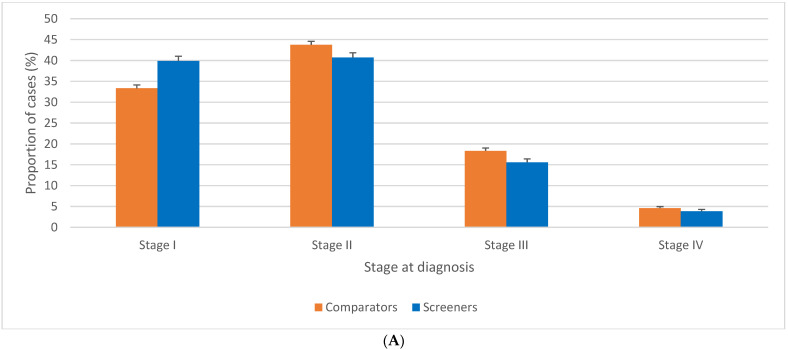

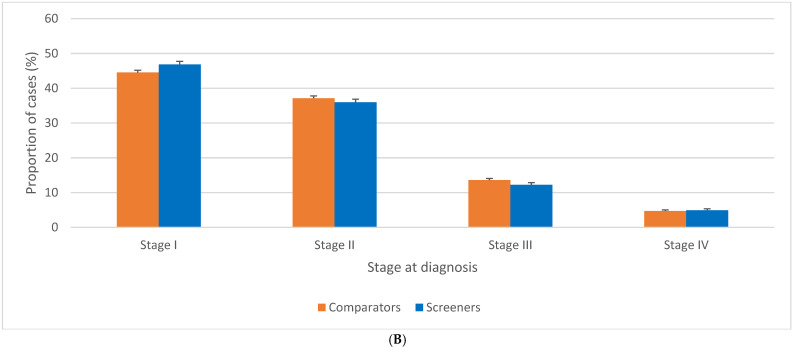
(**A**). Stage-specific distribution of female breast cancer cases by jurisdictional screening status, ages 40 to 49 years, Canada excluding Quebec, 2010 to 2017. (**B**). Stage-specific distribution of female breast cancer cases, ages 50–59 years, by jurisdictional screening status for women 40–49 years, Canada excluding Quebec, 2010 to 2017. Note: Quebec is excluded because cases diagnosed in Quebec from 2011 onward had not been submitted to the Canadian Cancer Registry. Screeners: Alberta, British Columbia, Nova Scotia, Northwest Territories, and Prince Edward Island; Comparators: Manitoba, New Brunswick, Newfoundland and Labrador, Nunavut, Ontario, Saskatchewan, Yukon. Source: Canadian Cancer Registry (1992 to 2018) at Statistics Canada [42,44]; provincial and territorial screening practices [49,50].

BC stage distribution at diagnosis in women aged 40–49 was significantly different in screener versus comparator jurisdictions (Figure 6A). Higher proportions of stage I BC were diagnosed among screener jurisdictions (33.3% vs. 39.9%, *p* < 0.001), while comparators had proportionately more BC diagnosed at stage II (43.7% vs. 40.7%, *p* < 0.001), III (18.3% vs. 15.6%, *p* < 0.001) and IV (4.6% vs. 3.9%, *p* = 0.001). Among the BC cases that were coded as unstaged, 96.9% were diagnosed in the comparator province of Ontario (data not shown). As a consequence, there were significantly more unstaged BC cases among comparators than screeners, with an incidence rate ratio of 2.4 (data not shown).

**Figure 7:**
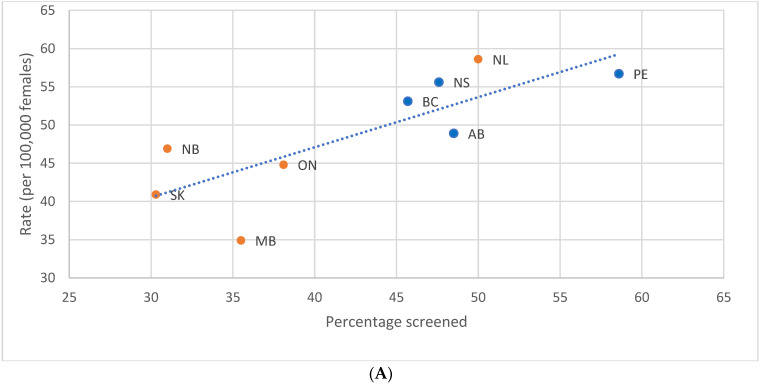

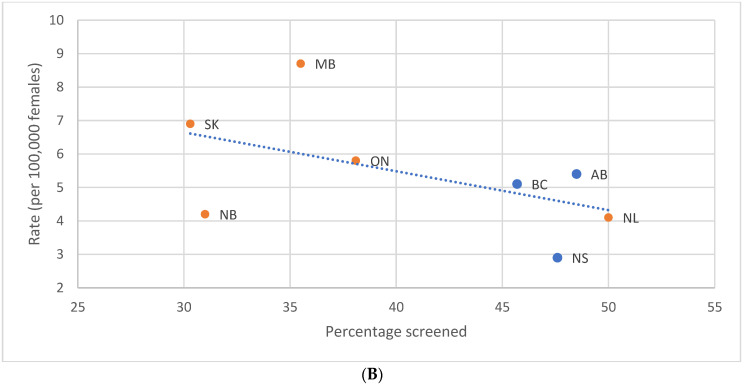
(**A**). Incidence rate of stage I female breast cancer during the 2011 to 2013 period by provincial screening participation rate in 2012, ages 40 to 49 years, selected provinces. (**B**). Incidence rate of stage IV female breast cancer during the 2011 to 2013 period by provincial screening participation rate in 2012, ages 40 to 49 years, selected provinces. Note: Orange dots denote comparators and blue dots denote screeners. Quebec is excluded because cases diagnosed in Quebec from 2011 onward had not been submitted to the Canadian Cancer Registry. The three Canadian territories (Yukon, Northwest Territories, and Nunavut) were excluded from the stage I and IV analyses, and Prince Edward Island from the stage IV analysis, because the number of incident cases was too small to be reliably compared against other jurisdictions. Source: Canadian Cancer Registry (1992 to 2018) and Canadian Community Health Survey [42,44]: Annual Component (2012) at Statistics Canada; provincial and territorial screening practices [49,50].

Regression of provincial and territorial screening participation with an incidence of the stage at diagnosis revealed a significant relationship between screening and stage I BC (*p* = 0.010). An increase of 10 percentage points in the screening participation of women aged 40–49 was linearly associated with a 6.6 per 100,000 increase in the stage I incidence rate (Figure 7A) and with a non-significant 1.2 per 100,000 decrease in the rate of stage IV or metastatic disease (*p* = 0.186) (Figure 7B).

### 3.5. Impact of Provincial/Territorial Screening Status for Women Aged 40–49 on BC Stage at Diagnosis in Women Aged 50–59

The stage distribution of BC cases diagnosed among women aged 50–59 differed according to the screening practices—screener or comparator—used in their jurisdiction for 40–49 year-olds (Figure 6B). There was a lower proportion of stage I in women aged 50–59 at diagnosis (44.5% vs. 46.8%, *p* < 0.001), and higher proportions of stage II (37.2% vs. 36.0%, *p* = 0.003) and stage III (13.6% vs. 12.3%, *p* < 0.001) in the comparator than in the screener jurisdictions. No significant difference was observed between comparator and screener jurisdictions in terms of the proportion of cases diagnosed at stage IV.

## 4. Discussion

This study leveraged differences in provincial and territorial screening practices and used robust Canadian BC data to investigate the impact of mammography screening programs on women aged 40–49 and 50–59. Even with suboptimal screening participation, a stage shift was noted in jurisdictions without organised screening programs. A significant relationship between the degree of jurisdictional screening participation and the incidence rate of stage I BC at diagnosis was observed. On a proportionate basis, women aged 40–49 in comparator provinces were significantly more likely to be diagnosed with stages II, III, or IV BC than their screened peers. For the first time, it was observed that Canadian screening programs that included women in their 40s were associated with earlier stage migration in women aged 50–59. In jurisdictions where women in their 40s were not included in the screening programs, there were significantly higher rates of stages II and III BC in women aged 50–59, and a significant increase in the incidence of metastatic BC over time. The stage profile of BC was observed to have changed since 2011 when the CTFPHC recommended against screening women aged 40–49. Changes appeared more evident among women aged 40–49 than among women aged 50–59 for whom screening continued to be recommended. The ongoing screening activity, albeit lessened, for women aged 40–49 may have mitigated the extent of this shift. 

These findings have implications for outcomes for these women, as stage III and IV BC have an overall five-year net survival of only 74.0% and 23.2% compared to 91.9% and 99.8% for stage II and I, respectively [54]. The profile of BC in women in their 40s is often of later stage (II, III, and IV) disease at diagnosis, compared to their screened peers in their fifties. This late-stage disease, along with the increased proportion of stage III BC in women aged 50–59, results in higher mortality and life years lost, given the young age at diagnosis and lower stage-specific survival [38]. In addition to the inherently increased mortality risk with later-stage disease, these women must also undergo treatments that are far more extensive than those for early BC. A diagnosis with late-stage BC often means more invasive surgeries such as mastectomy and axillary dissection, and more intensive and longer duration of therapy with chemotherapy and radiation, causing morbidity with potential long-term toxicities such as lymphedema, secondary malignancies, and cardiotoxicity [55]. 

The cost of annual mammography screening for women aged 40 to 49 has been estimated at CAD 2355 per woman [56]. However, these new costs would only be applicable to those women not already undergoing screening. They could also be partially offset by the reduction in the incremental costs of otherwise having to treat more stage II, III, and IV BC with increasingly complex and expensive therapies. In Ontario in 2014, the two-year treatment costs for stage I through IV BC were CAD 29,938; CAD 46,893; CAD 65,369, and CAD 66,627, respectively [57]. The treatment of hormone receptor-positive (HR+)/human epidermal growth factor 2 negative (HER2-) metastatic disease in Ontario from 2012 to 2017 cost more than CAD 1.2 billion [58]. These costs likely underestimate the current financial impacts of treating advanced BC. A two-year time frame does not capture all costs of stage III treatment, including recurrence and ongoing survivorship care, nor the full duration of treatment for metastatic disease. As well, these costs predate the introduction of cyclin-dependent kinase (CDK) inhibitors in metastatic BC, drugs which cost more than CAD 5000 per month [59] and can be continued for more than five years [60]. These costs also do not account for the loss of productivity from time away from the workforce, due to the lengthier treatment of more advanced cancers. The high costs of implementing a population-based screening program for all Canadian women 40–49 might be offset by a risk-stratified approach; research underway may provide greater insights into the best screening approach [61].

Several reasons for not screening women in their 40s have been cited: overdiagnosis, lead time bias, and the presence of more aggressive molecular subtypes which present with more advanced disease and have a higher risk of recurrence and death [62,63]. A recent analysis, after adjusting for tumour subtype and detection method (screening or diagnostic) showed no difference in survival for women aged 40–50 compared to women aged 51–60 [64]. This finding supports diagnosing breast cancers at an early stage in women aged 40–49, perhaps even more so if the cancers are aggressive. Overdiagnosis is more likely to be a concern in older populations who have higher rates of co-morbidities and is less likely to impact younger women with few underlying medical issues [38]. Overdiagnosis has been estimated to be <0.1% in women aged 40–49 [4]. Our study supports that cancers diagnosed in women in their 40s are not overdiagnosed, as higher proportions of stage III and metastatic disease in unscreened women suggest progression of the earlier stage BC diagnosed in screened women. An additional downside of breast cancer screening is that many women who are screened are never found to have breast cancer. Because 80% of women who develop breast cancer have no identifiable risk factors, a risk-based approach to limiting screening to only those at high risk has not been beneficial. Active research on strategies to evaluate a personalised approach to screening may include more intensive supplemental screening for some and reduced screening intensity for others [61].

Just as the absence of ethnicity in Canadian cancer data does not allow us to analyse BC outcomes related to race, current screening guidelines may not account for our racially diverse society. Screening guidelines are largely based on eight randomised controlled trials performed 30–60 years ago in Sweden, Scotland, the USA, and Canada [19]. Ethnicity was not recorded but given each country’s population, it is likely that white women were mainly studied. The biology of BC differs based on ethnicity. The incidence of BC in White women peaks in their 60s, while the highest incidence for Black, Hispanic, and Asian women is in their 40s [65,66]. Higher mortality rates in Black women are independent of socioeconomic factors and are driven by higher rates of triple-negative BC, an aggressive cancer that is associated with later stage presentation and resultant lower survival [67,68,69]. Current screening guidelines amplify disparities, as the requirement for self-referral, or need for primary care referral for screening, decreases screening uptake, and skews participation to those patients who are health-literate and of a higher socioeconomic background [36]. Initiating breast screening at the age of 40 has been shown to reduce the mortality disparities between Black and White women and create a more equitable screening strategy [70]. 

This study has several limitations. It is a retrospective analysis of the impact of jurisdictional screening program policies. Quebec, the second largest jurisdiction in Canada, had to be excluded as their BC cases had not been submitted to the CCR for diagnosis years 2011 onward. None of the screener jurisdictions had optimal participation of >70%, and many of the comparator provinces had some screening activity, including greater than 50% in Newfoundland and Labrador. As such, our analysis likely underestimates the impact of organised screening programs. Among women aged 40–49, the five-year net survival estimates for unstaged and unknown stage BC cases are intermediate to that of stage II and III cases, and a few percentage points below stage I to IV combined [54]. Definitively staging these cases likely would have led to slightly higher proportions of late-stage disease in both screener and comparator groups, but more so in the comparator group given the higher rate of unstaged cases in this group. Although the CCHS survey questions attempt to measure screening mammograms only, we cannot definitively determine if the mammograms reported were diagnostic or for screening purposes. The inclusion of diagnostic mammograms in this study could overestimate late-stage or symptomatic, palpable cancers in the screener jurisdictions, thereby further attenuating the impacts of screening. Although the aim of screening is to detect cancers at an early stage, we did not have information about how the BC was diagnosed, and some cancers may have been detected symptomatically or as interval cancers. The power to detect trends in women in their 40s and 50s, especially with sub-analyses by screening status, was limited by the fact that we only have seven years of stage data. Because this study only evaluated invasive carcinomas, the impact on in situ disease was not assessed. Risk factors could not be assessed in this study and it is possible that 40–49-year-old women had more risk factors in the comparator than in the screening groups. However, given that we measured the stages of breast cancer at diagnosis this was unlikely to have significantly impacted the outcomes. In addition, the Canadian database did not record race or ethnicity, or breast tissue density, and we could not assess the impact of screening guidelines on the groups who would most likely be affected, such as Black and Asian women whose incidence of breast cancer peaks in the 40s, or those at higher risk. While the use of national registry data stands out as one of the main strengths of this study, the absence of statistical information on cancer recurrence and longer-term outcomes after an initial BC diagnosis narrows the scope of our conclusions to primary tumour cases. We were unable to evaluate breast cancer mortality outcomes using 10-year follow-ups for cases diagnosed from 2010–2017 as follow-up was restricted to the end of 2017.

This study presents important new information regarding the impact on Canadian women in their 40s and 50s of current BC mammography screening guidelines. The results can be used to reassess the optimum lower age for BC screening in Canada. Although women aged 40–49 have a lower incidence of breast cancer than those 50–59, women in both these age groups may have been negatively impacted by the exclusion of women younger than 50 from organised breast screening programs. This study supports two large observational trials [20,21], showing the benefit of breast cancer mortality reduction among women 40 years and older. Future research into the best method of including women 40–49 years old in population-based screening programs is needed. The thousands of Canadian women in their 40s who are diagnosed with BC each year have proportionally more stage III and metastatic cancers than women involved in organised screening, and there may be downstream stage migration with its associated decreased stage-specific survival in women aged 50–59. Identifying these cancers through screening at an early stage, where associated treatment costs are reduced relative to late-stage disease, could result in substantial savings for the Canadian health care system. The current screening guidelines are inequitable because they necessitate self-referral, selecting for health-literate women of higher socioeconomic status, and creating barriers for women who are required to have a primary care provider who will agree to the referral. These same guidelines preferentially benefit White women while disadvantaging ethnic groups with differing BC biology with an earlier peak incidence. Our guidelines may lead to increased treatment morbidity, and most importantly increased mortality. It is time to focus not on the harms of screening, but on the harms of not screening women aged 40–49. 

## 5. Conclusions

Between 2010 and 2017, using national database registries, Canadian women 40–49 years old were diagnosed with significantly fewer stage I and more stages II and III breast cancers than women 50–59 years old. In the same period, women 40–49 years old in screening jurisdictions were diagnosed with significantly fewer stages II, III, and IV BC than those living in the comparator provinces. Screening policies for women 40–49 also had an impact on women 50–59 years old who had significantly lower rates of stages II and III BC in the screening vs. the comparator jurisdictions.

## Figures and Tables

**Table 1:** Breast cancer screening information and participation rates by province and territory, women aged 40–49, Canada, selected years.

Province/Territory	Screening Programmatic Information (2007–2008)		Screening Participation Rates			
	Referral 40–49	Recall	2003	2008	2012	2017
British Columbia *	Self	Annual	44.2	47.4	45.7	39.0
Alberta **	Self	Annual	43.2	52.9	48.5	44.1
Saskatchewan	No	None	26.6	27.9	30.3	32.6
Manitoba	MD	Biennial	24.5	21.8	35.5	18.1
Ontario	MD-High Risk only	High Risk-Annual	33.1	37.5	38.1	27.0
Quebec	MD	None	26.0	31.4	25.2	21.4
New Brunswick	MD	None	42.2	41.4	31.0	20.8
Nova Scotia	Self	Annual	50.2	50.7	47.6	45.5
Prince Edward Island	Self	Annual	31.2	35.2	58.6	45.4
Newfoundland and Labrador	No	None	41.9	51.8	50.0	51.3
Northwest Territories	Self	Annual	43.9	42.7	63.6	n/a
Yukon	Self	None	10.0	32.6	11.4	n/a
Nunavut	n/a	n/a	12.2	13.2	41.0	n/a

n/a = no screening data available. Note: screening percentage rates refer to the percentage of women 40–49 with a screening mammogram in the previous two years, based on data from the Canadian Community Health Survey. Shaded provinces and territories denote screeners while the non-shaded ones are the comparators. Source: Canadian Community Health Survey, Cycle 2.1 (2003), Annual Component (2008, 2012, 2017); provincial and territorial screening practices [49,50]. Screening program information changed in some jurisdictions throughout the study period: * BC changed from annual to biennial recall in 2014. ** Alberta changed from self-referral to requiring an MD referral for the first screen in 2012.

**Table 2:** Number and proportion of women diagnosed with breast cancer by stage at diagnosis, ages 40 to 49 years versus 50 to 59 years, Canada excluding Quebec, 2010 to 2017 period.

Stage at Diagnosis	40 to 49 Years			50 to 59 Years			*p*-Value for Differences in Stage-Specific Proportions
	Number	Proportion of Cases Diagnosed at Stages I to IV (%)	95% CI of the Proportion	Number	Proportion of Cases Diagnosed at Stages I to IV (%)	95% CI of the Proportion	
Stage I	7200	35.7	(35.0, 36.3)	15,125	45.3	(44.8, 45.8)	<0.001
Stage II	8600	42.6	(41.9, 43.3)	12,265	36.7	(36.2, 37.3)	<0.001
Stage III	3500	17.3	(16.8, 17.9)	4385	13.1	(12.8, 13.5)	<0.001
Stage IV	880	4.4	(4.1, 4.7)	1610	4.8	(4.6, 5.1)	0.005
Unknown	140	NA	NA	210	NA	NA	NA
Unstaged	645	NA	NA	930	NA	NA	NA
Total	20,965	NA	NA	34,525	NA	NA	NA

NA = not applicable, CI = confidence interval. Note: Quebec is excluded because cases diagnosed in Quebec from 2011 onward had not been submitted to the Canadian Cancer Registry. Counts have been randomly rounded to a multiple of five in accordance with Statistics Canada’s disclosure-avoidance guidelines. Source: Canadian Cancer Registry (1992 to 2018) at Statistics Canada [42].

## Data Availability

Aggregate data on cancer incidence counts and rates by stage are publicly available from Tables 13-10-0761-01 and 13-10-0762-01, patient- and tumor-level microdata files are confidential and can only be accessed through Statistics Canada’s Research Data Centre Program.
